# Study of Temperature Characteristics of Micromachined Suspended Coplanar Waveguides for Biosensing Applications

**DOI:** 10.3390/s110302640

**Published:** 2011-03-01

**Authors:** Lijie Li, Zhuming Liu

**Affiliations:** Multidisciplinary Nanotechnology Centre, College of Engineering, Swansea University, Swansea, SA2 8PP, UK

**Keywords:** temperature characteristics, coplanar waveguide, biosensors

## Abstract

In the recent development on biosensors, coplanar waveguide based microwave dielectric sensors have been attracting more and more attentions. In this paper, microwave performance of a suspended coplanar waveguide subject to temperature variations, particularly in a small range, is studied. The prototype is realized through a MEMS fabrication foundry. The thermal transfer analysis of the device is conducted using finite element method, and the microwave properties of the device are characterized. One of the results shows that at 20 GHz, the S11 has decreased by 7.4%, and S21 has increased by 3.5% when the voltage applied to the heaters varies from 9 V to 29 V.

## 1. Introduction

Microwave biosensors usually don’t require the biosamples to be optically or chemically altered, which is a big advantage compared with optical and chemical biosensors. Micromachined coplanar waveguides have been used to realize biosensors due to their smaller size and high performance [1,2]. Several examples of using coplanar waveguides as microsensors are summarized as follows. A gas sensor composed of a suspended micromachined coplanar waveguide with carbon nanotubes as dielectric materials was reported in [3], which was based on gas-induced variations in dielectric permittivity of carbon nanotubes. Demonstration of a Gaubau transmission line for biosensor applications has been reported in [4], which was constructed on a coplanar waveguide. A wide-bandwidth, high-sensitivity particle sensing and cell counting device in a microfluidic system was presented using coplanar waveguide technology [5]. Distributed transmission lines have been utilized to serve as biosensors, which have the advantage of intensified interactions between electromagnetic waves and biosamples in slow-wave structures [6–8]. In the biosensing applications, it is quite often that the temperature of biosamples changes due to chemical reactions or electrical heating from the sensor itself. Thus study of temperature characteristics of coplanar waveguide is important to understand the impact on microwave performances of the sensors upon temperature variations. It is known that high temperature will have some effects on the performance of the coplanar waveguides, as the series resistance of the signal line varies due to temperature variations [9]. Many previous theoretical and experimental studies described temperature-dependence of the traditional coplanar waveguides [10–12]. However most of the above investigations were based on evenly temperature rising of the CPW structures, or based on self-heating by the RF signal passing through the CPW [13,14]. In many real cases, the temperature rising happens just in parts of the CPW structure where the heat sources are some adjacent electronic circuits. To the best of author’s knowledge, there is no previous scientific literature reported on investigation of the CPW performance under external localized heating.

In this paper, a new structure that consists of a suspended CPW and two silicon heaters adjacent to the ground plane has been designed for the purpose of investigating microwave performances of the CPW subject to the localized heating. The structure has been fabricated through a MEMS fabrication process and subsequently characterized using microwave equipments. Temperature profiles of the heaters and CPW have been modeled using a finite element software. The paper is structured as follows: section 2 of the paper describes the design, fabrication, and thermal transfer modeling of the device. Microwave measurements of the device are reported in section 3. Finally in section 4 some conclusion remarks are made.

## 2. Design, Fabrication, and Thermal Modeling of the Device

**Figure 1.**
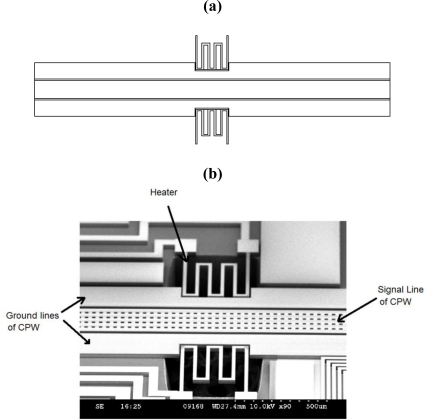
**(a)** Schematic graph of the device. **(b)** SEM graph of the device.

In order to study the temperature impact on the CPW caused by surrounding heat sources. A test structure that consists of a suspended CPW and two suspended spring-shaped silicon heaters located on both sides of the CPW symmetrically has been designed. The schematic graph of the test structure is shown in Figure 1(a). In principle, heat is generated by joule heating of the two silicon resistors through which electrical current flows, subsequently temperature of the ground plane of the CPW will increase through heat transfer mechanisms, and the temperature of the signal line of CPW increases as well. However due to the localized heating, the temperature distributions along the ground plane and signal line of the CPW are not the same. Prototype of the test structure has been realized through silicon-on-insulator based MEMS foundry process. The fabrication procedure is summarized as follows: a silicon-on-insulator wafer is prepared and the silicon layer has been patterned. Next, the surface of the silicon layer has been metalized for the purpose of creating electrical conductors and increasing optical reflectivity of the surface. In this case, metallization is used for increasing the conductivity of the coplanar waveguide. The heater structure is not coated with the metal. Finally the wafer has been back-etched from the bottom side, so that the structures in the silicon layer can be released. This step is used to realize the suspended CPW and heaters. The fabricated structures have been examined by the scanning electron microscope and Figure 1(b) shows one of the SEM images. Small holes are designed in the signal line to reduce the initial stress during the releasing process.

**Figure 2.**
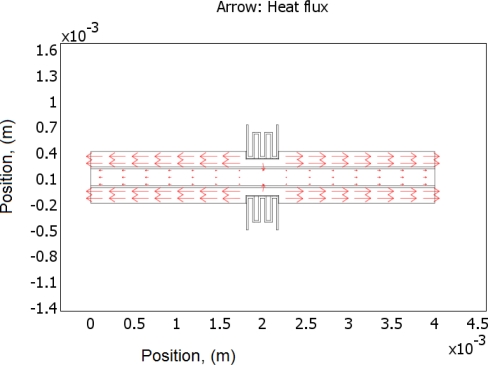
Simulation result of heat flux through CPW.

**Figure 3.**
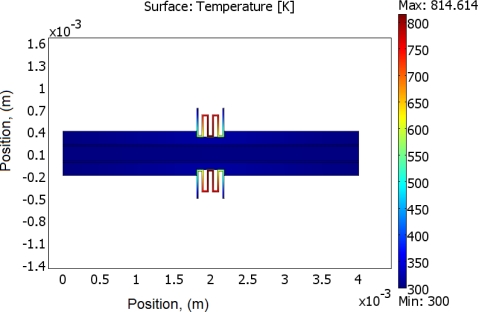
Simulated temperature distribution in the device.

**Figure 4.**
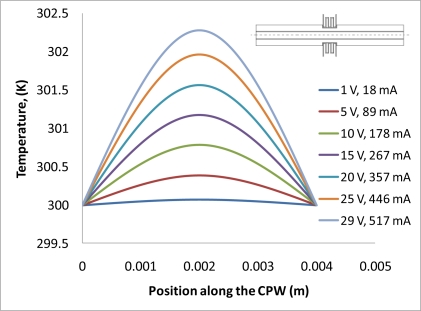
Temperature distribution in the signal line of CPW.

**Figure 5.**
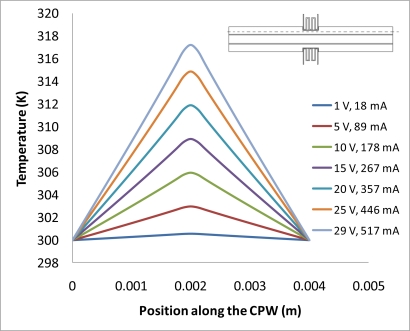
Temperature distribution in the ground line of CPW.

Since the structure is very small, it is very difficult to measure its temperature using thermometers. Theoretical analysis of the temperature distribution has been conducted based on the finite element method (FEM); here a FEM software was used to perform the electrical-thermal transfer analysis. Joule heating in microdevice is a well known phenomenon that has been studied extensively, particularly when people were looking at electrothermal actuators [15,16]. One dimensional analytical thermal modelling can be used to simulate structures that have uniform width and thickness, for example in [15]. However for thermal transfer problem in complex structures, such as for the case reported in this paper, two-dimensional thermal transfer analysis has to be performed using finite element method. For the spring-shaped heater structure in the Figure 1, the temperature of the heater itself and different part of the CPW is governed by the heat conduction transfer equation:

(1) $$-\nabla \cdot (k\nabla T)=Q+h_{\mathrm{trans}}(T_{\mathrm{ext}}-T)+C_{\mathrm{trans}}(T_{\mathrm{amtrans}}^{4}-T^{4})$$

where T is the temperature value in the structure, *h_trans_* is the convective heat transfer coefficient, Temperature, *T_ext_* is the external temperature, and *T_ambtrans_* is the ambient temperature, and *C_trans_* is the user defined constant for the radiation thermal transfer. In this case, firstly heat is generated by joule heating when electrical current passes through the spring shaped structure, and it is described as

(2) $$-\nabla \cdot d(\delta \nabla V-J^{e})={\mathrm{dQ}}_{j},\sigma =1/(\rho_{0}(1+\alpha (T-T_{0})))$$

where *J^e^* is the external current density, *Q_j_* is the current source, *d* is the thickness of the structure, *ρ*_0_ is the resistivity at reference temperature, *α* is the temperature coefficient, *T*_0_ is the room temperature. Next, the generated heat is partially transferred through the air gap into the ground line of CPW, and further into the signal line. Finite element software, COMSOL, is used to solve this problem based on above equations. Geometric and physical parameters that were used for the simulations are shown in Table 1. In this simulation, radiation is neglected, since the simulated temperatures are mostly below 800 K [17]. Figure 2 schematically shows the direction of heat transfer in this device. The length of the arrow shown in the graph stands for the quantity of the heat flux. It is shown that much more heat has been transferred into the ground plane than into the single line, since there is an air gap (20 μm) between ground line and the signal line, and the thermal conductivity of the air is much less than that of the silicon. In Figure 3, a simulation result shows the temperature distribution of whole structure when there is 29 volts being applied to both the heaters. It is seen that although the temperature of the heater itself is very high (maximum temperature point is around 814 K), temperatures in CPW has not been much affected. That is due to the fact that a thin air gap (6 μm) between heater and ground line of CPW, and another air gap (20 μm) between ground plane and single line. Figure 4 shows the simulated results of the temperature distribution in the centre of the CPW signal line. DC voltages ranging from 1 V to 29 V have been applied to the heaters, and the maximum calculated temperature in the CPW signal line is around 302 K, which is only 2 degrees higher than the ambient temperature. However looking at the Figure 5, which shows the temperature distribution along the CPW ground line, the calculated temperatures are much higher. Around 318 K is simulated when the voltage is 29 V. Figures 4 and 5 also show the calculated electrical current at every applied voltage value. From Figures 4 and 5, we can see that shapes of temperature distributions are different, one has a parabolic shape, and the other has a triangular shape.

## 3. Microwave Measurements

**Figure 6.**
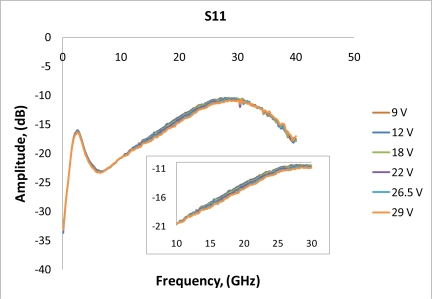
Measurements of reflection coefficient S11 for different applied voltages into the heaters.

**Figure 7.**
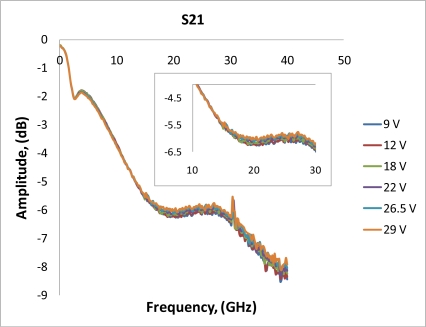
Measurements of transmission coefficient S21 for different applied voltages into the heaters.

**Figure 8.**
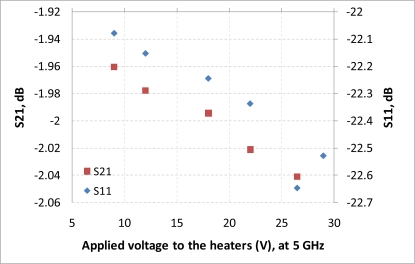
scattering parameters *vs.* applied voltage to the heaters, f = 5 GHz.

**Figure 9.**
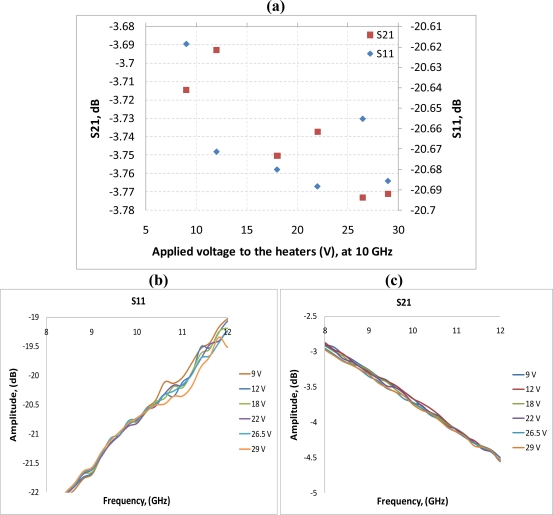
**(a)** scattering parameters *vs.* applied voltage to the heaters, f = 10 GHz; **(b)** S11 in the range of 8GHz to 12 GHz; **(c)** S21 in the range of 8 GHz to 12 GHz.

**Figure 10.**
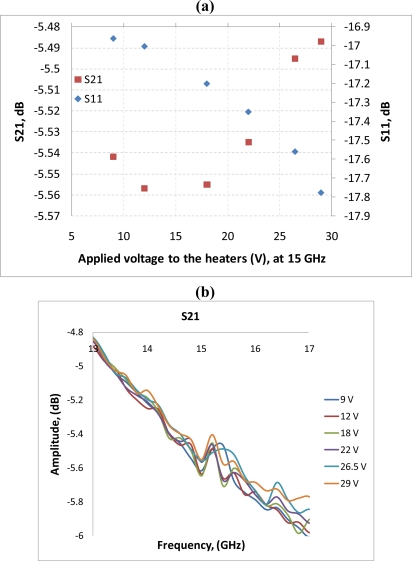
**(a)** scattering parameters *vs.* applied voltage to the heaters, f = 15 GHz; **(b)** S21 in the range of 13 GHz to 17 GHz.

**Figure 11.**
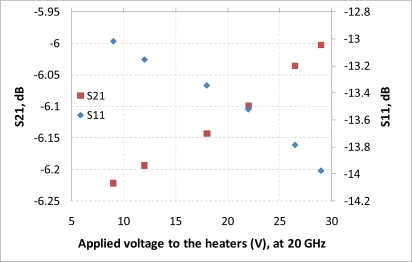
scattering parameters *vs.* applied voltage to the heaters, f = 20 GHz.

**Figure 12.**
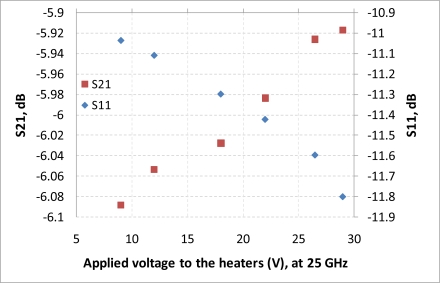
scattering parameters *vs.* applied voltage to the heaters, f = 25 GHz.

**Figure 13.**
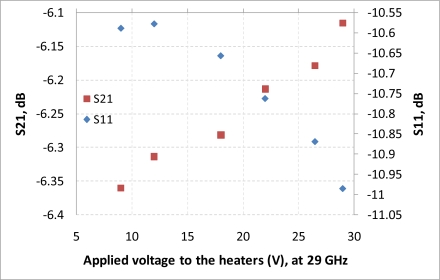
scattering parameters *vs.* applied voltage to the heaters, f = 29 GHz.

**Figure 14.**
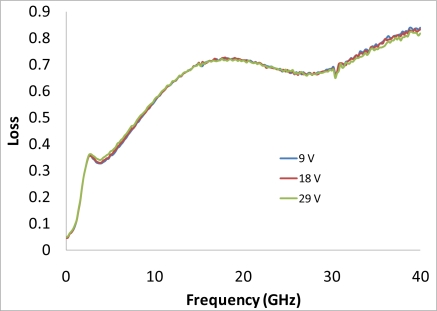
losses of the suspended transmission line *vs.* applied voltages.

After performing simulations of temperature distributions of CPW due to localized heating, the device has been measured using a vector network analyzer (VNA) and a coplanar probe station. Before doing measurements, the probe station was calibrated using standard substrates. Applying voltages through the heaters, scattering parameters of the CPW have been recorded. Figures 6 and 7 display the measured results. It is seen that even for such small temperature variations predicted by the simulation in the last section, the reflection coefficient (S11) and transmission coefficient (S21) of the CPW have changed. The S11 has changed from −13.02 dB to −13.98 dB at 20 GHz when the voltage applied to the heaters has varied from 9 V to 29 V, which corresponds to the maximum temperature of the CPW ground line changing from around 306 K to 317 K. Under the same experiment conditions, S21 has changed from −6.22 dB to −6.01 dB. The relationships between measured scattering parameters and applied voltages to the heaters at 5 GHz, 10 GHz, 15 GHz, 20 GHz, 25 GHz, and 30 GHz are shown in Figures 8–13 respectively. As there is relatively large noise in the measurement compared to the signal level, average values (in the range of ±0.5 GHz) of S11 and S21 have been taken at above frequency points. It is shown from these figures, that reflection coefficient S11 has a decreasing trend with increasing temperatures except at 10 GHz (Figure 9 (a)). We have further investigated S parameters at around 10 GHz and found that it is very difficult to see how microwave performances vary against temperatures as the noise level is higher than the signal level (shown in Figure 9(b,c)). For S21, it decreases when the microwave signal at 5 GHz and 10 GHz (again the trend of S21 cannot be seen very clearly due to noise), and this trend has changed reversely when the microwave signal exceeds 15 GHz. It is seen that at 15 GHz, S21 decreases when voltages are below 15 V, and increases for voltages above 15 V. To further study microwave performance at this frequency, S21 in frequency range of 13 GHz to 17 GHz has been plotted in Figure 10(b), It is seen that the noise is too large compared to the signals, therefore the trend of S21 at around 15 GHz is not clear. In terms of amplitude of the scattering parameters, it shows that S11 increases linearly with increasing temperatures. At 5 GHz, the amplitude of S11 has changed by 2.1% when the applied voltage to the heaters has varied from 9 V to 29 V, and the amplitude of S21 has changed by 5.5%. For 15 GHz, the amplitude variation of S11 is 4.8%. When the microwave frequency is at 20 GHz, the S11 and S21 amplitudes have changed by 7.4% and 3.5%. While at 25 GHz, S11 and S21 amplitudes have changed by 6.9% and 2.8%. Finally at 30 GHz, the amplitudes of S11 and S21 have changed by 3.7% and 3.8%. For the current design, the temperature variations in the CPW structure are very small according to the heat transfer simulation in section 2. The temperature of the CPW cannot be further increased in this study, because the maximum temperature of the heaters almost reaches the point that causes thermal damage of the silicon. According to microwave theory, if the network has no loss and no gain, the output power must equal to input power, and so in this case |*S*11|^2^ + |*S*21|^2^ = 1. In order to find out the overall losses of the CPW *versus* temperatures, the losses (1 – |*S*11|^2^ − |*S*21|^2^) have been calculated and shown in Figure 14. It is seen from the Figure 14 that in low frequency range (0–14 GHz) losses increase with rising temperatures, and at high frequencies, losses decrease with rising temperatures.

## 4. Conclusions

Study of temperature effects on the RF performances of suspended CPW has been conducted using a test structure that consists of a suspended CPW and two symmetric spring-shaped silicon heaters adjacent to the CPW. Thermal transfer between the heaters and CPW has been simulated using FEM software, and the results show that although the maximum temperature of the silicon heaters is very high, the temperature of the CPW has not been much increased due to an air gap between heaters and CPW. Microwave measurements of the device have also been conducted, it is concluded that at low frequency band (<15 GHz), the scattering parameters have changed in much lower amplitude than at higher frequency band (>15 GHz). The experiments also show that S11 has a decreasing trend with increasing temperatures except at frequency point of 10 GHz, while S21 is reversely proportional to the temperature at lower frequency band, and becomes proportional to the temperature at higher frequency band. In the future, customized micromachining process will be selected in order to have the heaters directly in contact with the ground plane of the CPW, so that the effects on RF performances of the suspended CPW at much higher temperatures can be obtained.

## Figures and Tables

**Table 1.** Geometric and physical parameters in the simulation.

Parameter	Value	Unit
Thickness of structure	20	μm
Length of CPW	4,000	μm
Width of CPW ground line	180	μm
Width of CPW signal line	200	μm
Total length of heater	2,000	μm
Width of heater	20	μm
Air gap between heater and CPW ground line	6	μm
Air gap between ground line and signal line of CPW	20	μm
Resistivity of silicon	5.6 × 10^−6^	Ω-m
Temperature coefficient	1	1/K
Ambient temperature	300	K
Thermal conductivity of silicon	130	W/(m*K)
Thermal conductivity of air	0.03	W/(m*K)
Convective heat transfer coefficient	10	W/(m^3^*K)
External temperature	300	K
